# Characterization of immunization secondary analyses using demographic and health surveys (DHS) and multiple indicator cluster surveys (MICS), 2006–2018

**DOI:** 10.1186/s12889-021-10364-0

**Published:** 2021-02-12

**Authors:** Yue Huang, M. Carolina Danovaro-Holliday

**Affiliations:** 1 Department of Immunization, Immunization, Analytics and Insights (IAI), Vaccines and Biologicals (IVB), World Health Organization (WHO), 1211 Geneva, Switzerland; 2 Present affiliation: State Key Laboratory of Molecular Vaccinology and Molecular Diagnostics, National Institute of Diagnostics and Vaccine Development in Infectious Diseases, Strait Collaborative Innovation Center of Biomedicine and Pharmaceutics, School of Public Health, Xiamen University, Xiamen, 361102 China

**Keywords:** DHS, MICS, Immunization coverage, Secondary analysis, Survey

## Abstract

**Background:**

Infant immunization coverage worldwide has plateaued at about 85%. Using existing survey data to conduct analyses beyond estimating coverage may help immunization programmes better tailor strategies to reach un- and under-immunized children. The Demographic and Health Survey (DHS) and the Multiple Indicators Cluster Survey (MICS), routinely conducted in low and middle-income countries (LMICs), collect immunization data, yet vaccination coverage is often the only indicator reported and used. We conducted a review of published immunization-related analyses to characterize and quantify immunization secondary analyses done using DHS and MICS databases.

**Methods:**

We conducted a systematic search of the literature, of immunization-related secondary analyses from DHS or MICS published between 2006 and August 2018. We searched 15 electronic databases without language restrictions. For the articles included, relevant information was extracted and analyzed to summarize the characteristics of immunization-related secondary analyses. Results are presented following the PRISMA guidelines.

**Results:**

Among 1411 papers identified, 115 met our eligibility criteria; additionally, one article was supplemented by the Pan American Health Organization. The majority were published since 2012 (77.6%), and most (68.9%) had a first or corresponding author affiliated with institutions in high-income countries (as opposed to LMICs where these surveys are conducted). The median delay between survey implementation and publication of the secondary analysis was 5.4 years, with papers with authors affiliated to institutions in LMIC having a longer median publication delay (*p* < 0.001). Over 80% of the published analyses looked at factors associated with a specific vaccine or with full immunization. Quality proxies, such as reporting percent of immunization data from cards vs recall; occurrence and handling of missing data; whether survey analyses were weighted; and listing of potential biases or limitations of the original survey or analyses, were infrequently mentioned.

**Conclusion:**

Our review suggests that more needs to be done to increase the increase the utilization of existing DHS and MICS datasets and improve the quality of the analyses to inform immunization programmes. This would include increasing the proportion of analyses done in LMICs, reducing the time lag between survey implementation and publication of additional analyses, and including more qualitative information about the survey in the publications to better interpret the results.

**Supplementary Information:**

The online version contains supplementary material available at 10.1186/s12889-021-10364-0.

## Background

In past decades, great progress has been made in expanding the reach of immunization programme and introducing new vaccine s[1–3]. However, vaccine-preventable diseases (VPD) remain a significant cause of morbidity and mortalit y[4, 5].

Several initiatives have allied countries and immunization stakeholders towards reaching more people and preventing more vaccine-preventable diseases (VPDs). The Global Immunization Vision and Strategy 2006–2015 (GIVS )[6], then the Decade of Vaccines’ Global Vaccine Action Plan (GVAP )[4], and soon the Immunization Agenda 2030 (IA2030 )[7], have guided efforts of achieving the vision of delivering universal access to immunization. Yet, coverage levels worldwide have plateaued at about 85% for 3 doses of diphtheria-tetanus-pertussis vaccine (DTP3) and coverage gaps persist between countries, as well as within countrie s[8]. Thus, better understanding immunization program performance can help immunization programmes better tailor strategies to reach un- and under-immunized children [4].

Vaccine coverage (percentage of target population vaccinated) is a major indicator for immunization programme performance at the local, national, regional and global levels. It is used to target resources and identify areas requiring special attention, and is a core indicator for access to basic health service s[9]. Estimates of immunization coverage generally come from several sources, including administrative systems and registries and from different household survey s[6]. Household surveys are frequently used to complement administrative data, and in some cases as the primary measurement of vaccination coverage. Oftentimes, it is thought that household surveys can give the more accurate and precise estimates, although they have the disadvantage that they cannot provide timely enough information for program planning, and can be subject to several biase s[10–13]. The Expanded Programme on Immunization (EPI) cluster surve y[14], the United Nations International Children’s Emergency Fund (UNICEF) Multiple Indicator Cluster Survey (MICS) [15] and the Demographic and Health Survey (DHS )[16] are the three main household surveys used to obtain vaccination coverage.

DHS and MICS almost always include immunization-related questions and report coverage indicators. These coverage estimates are used at the global level to inform the World Health Organization (WHO) and UNICEF National Estimates of Immunization Coverage (WUENIC )[3] and to assess progress by other immunization partners like Gavi, the Vaccine Alliance [17]. Nevertheless, it is unclear how much the national immunization programs of countries where these surveys are implemented use DHS and MICS results, and especially whether secondary analyses on immunization are done, and if so, whether they are used to inform immunization program planning. To answer the first question, we conducted a review and characterization of immunization-related secondary analyses published in the literature between 2006, the year that GIVS was launched [6], and 2018.

## Methods

### DHS and MICS availability

We extracted information about DHS and MICS surveys done and availability of those survey databases (as they are needed to conduct secondary analyses) from their respective websites.

### Literature search

We searched articles, published between 2006 and August 2018, in 15 electronic bibliographic or full-text databases without language restrictions, including comprehensive databases from low and middle-income countries (LMICs) (Table 1).

**Table 1:** Database searching results

Database	Type of database	Characteristic	Result
Web of science (Web of science core collection)	Bibliographic	Comprehensive multiple databases.	487
PubMed	Bibliographic	Primarily the MEDLINE database.	168
PMC	Full-text	Publicly accessible full-text database.	241
The Institutional Repository for Information Sharing (IRIS)	Full-text	The digital library of WHO’s published material and technical information in full text produced since 1948. Its content is freely accessible and searchable in the six official languages (Arabic, Chinese, English, French, Russian & Spanish).	147
Index Medicus for South-East Asia Region (IMSEAR)	Bibliographic	A database of articles published in selected journals within the WHO South-East Asia Region.	114
Joanna Briggs Institute EBP Database	Full-text	Published by the Joanna Briggs Institute (JBI),a leading provider of evidence–based information.	91
CINAHL	Bibliographic	Database with journal articles about nursing, allied health, biomedicine and healthcare.	37
BLDS Index to development studies	Bibliographic	Europe’s most comprehensive research collection on development issues.	37
Directory of Open Access Journals Search	Bibliographic	Independent database contains ca. 12,000 open access journals covering all areas of science, technology, medicine, social science and humanities.	28
Popline	Bibliographic	Contains the world’s most comprehensive collection of population, family planning and related reproductive health and development literature.	26
Informit Health Collection	Full-text	An extensive range of authoritative subject-based databases featuring coverage of Australian and international information resources.	25
Western Pacific Region Index Medicus (WPRIM)	Bibliographic	Medical and health journals published in Member States of the WHO Western Pacific Region.	5
LILACS (Latin Am. & Carib. Center on Health Sci Info)	Bibliographic	Bibliographic database in the Health Sciences in Latin America and the Caribbean.	4
Cochrane	Bibliographic/ full-text	A collection of databases in medicine and other healthcare specialties provided by Cochrane and other organizations. At its core is the collection of Cochrane Reviews, a database of systematic reviews and meta-analyses.	1
African Journals online	Full-text	The world’s largest online library of peer-reviewed, African-published scholarly journals.	0

The searching strategy was: full text search of (DHS OR MICS OR “demographic and health survey” OR “demographic and health surveys” OR “demographic health survey” OR “demographic health surveys” OR “demographic & health survey” OR “demographic & health surveys” OR “multiple indicator cluster survey” OR “multiple indicator cluster surveys”). For databases with available MESH search, searching of (“Immunization”[Mesh] OR “Vaccination”[Mesh]) was combined. Otherwise, title and abstract searching of (Immun* OR Vaccin*) was done.

To avoid missing articles, we searched PubMed and PMC (PubMed Center) separately. For the Web of Science, we conducted both topic search and cited work search. All the searches were conducted under the technical guidance of the WHO librarian. The strategies were adapted to searching characteristics of each database. Staff from WHO regional offices were contacted to further identify missing articles.

The study protocol was submitted to PROSPERO. PROSPERO concluded that our study was a literature review with a systematic search, as opposed to a systematic literature review.

### Inclusion and exclusion criteria

The inclusion criteria were: 1) papers published on or after 2006; 2) the study was a secondary analysis from DHS or MICS (sub-national MICS were not excluded); and 3) the main topic in the study was immunization. All the articles identified were imported into Endnote for systematical assessment. The lead author (YH) independently conducted the initial literature screening. For the articles for which it was unclear if they met the inclusion criteria, the senior author (MCD-H) evaluated them and made the final decision regarding inclusion.

### Analyses

For the articles included, the information listed below was extracted and analysed:

1. Metrics related to the publication:

- Affiliation: for each article, the countries of all authors (based on their reported affiliations), and countries of first and correspondent authors were listed separately and analysed further. The countries were classified into high-income countries or LMICs according to 2018 World Bank classification [18].
- The year of survey conduction (last year if DHS or MICS expanded over two calendar years)
- The year of publication of the article.
- The population analysed (e.g., children aged 12–23 months, women of childbearing age).

2) Main content of the publication:

- Vaccine(s) included in the survey analysis.
- Outcomes: classification as complete or full immunization, vaccination with a specific vaccine(s) (e.g., measles-containing vaccine (MCV), diphtheria, tetanus and pertussis (DTP) vaccine, polio vaccine, Bacille Calmette-Guerin (BCG) vaccine, rotavirus vaccine, tetanus toxoid, hepatitis B vaccine, and so on), partial or incomplete immunization, and never vaccinated. If an article included multiple outcomes and various vaccines, they were all included and listed.
- Types of analyses done: these included exploring factors associated with the outcome; inequalities in vaccination, such socio-economic and geographic variability; timeliness (considered as having received particular vaccines within a certain timeframe of the recommended age); coverage trends; and “others”, such as supplementary immunization activities (SIA), mapping of immunization coverage, missed opportunities for vaccination (MOV), comparison of coverage estimates from multiple data sources, estimation of “effective vaccination coverage” using cross-sectional surveys combined with administrative data, immunization population targets, etc.
- Methodological approaches used for the analyses.

3) Inclusion of information related to “quality” in the article:

- Whether the study imputed missing data.
- Whether the study described how persons without documented vaccination evidence (i.e., without cards) were handled.
- Whether the study included percentage of documented vaccination seen for the target group of the survey.
- Whether the survey analysis was weighted.
- Whether the article listed potential biases in the original survey.
- Whether the article discussed potential limitations of the survey and/or the analysis.
- Whether the article included recommendations based on the findings, and if so, whether these recommendations met SMART criteria (specific, measurable, attainable, relevant, time-bound).

Chi-square test or Fisher’s exact test was used to analyse the difference of the distribution of variables in different groups. All statistical analyses were performed using SPSS (24).

## Results

As of August 2018, 308 standard DHS had been conducted (with 22 DHS ongoing at the time of the first writing in December 2018) in 91 low- and middle-income countries (LMICs) since 1985. Similarly, 352 MICS had also been done (with 60 not yet available) in 113 LMICs since 1993 (supplements 1,2) [19]. Regarding data publicly available, 94.1% (269/286) of DHS and 67.1% (196/292) of MICS datasets were available.

**Fig. 1:**
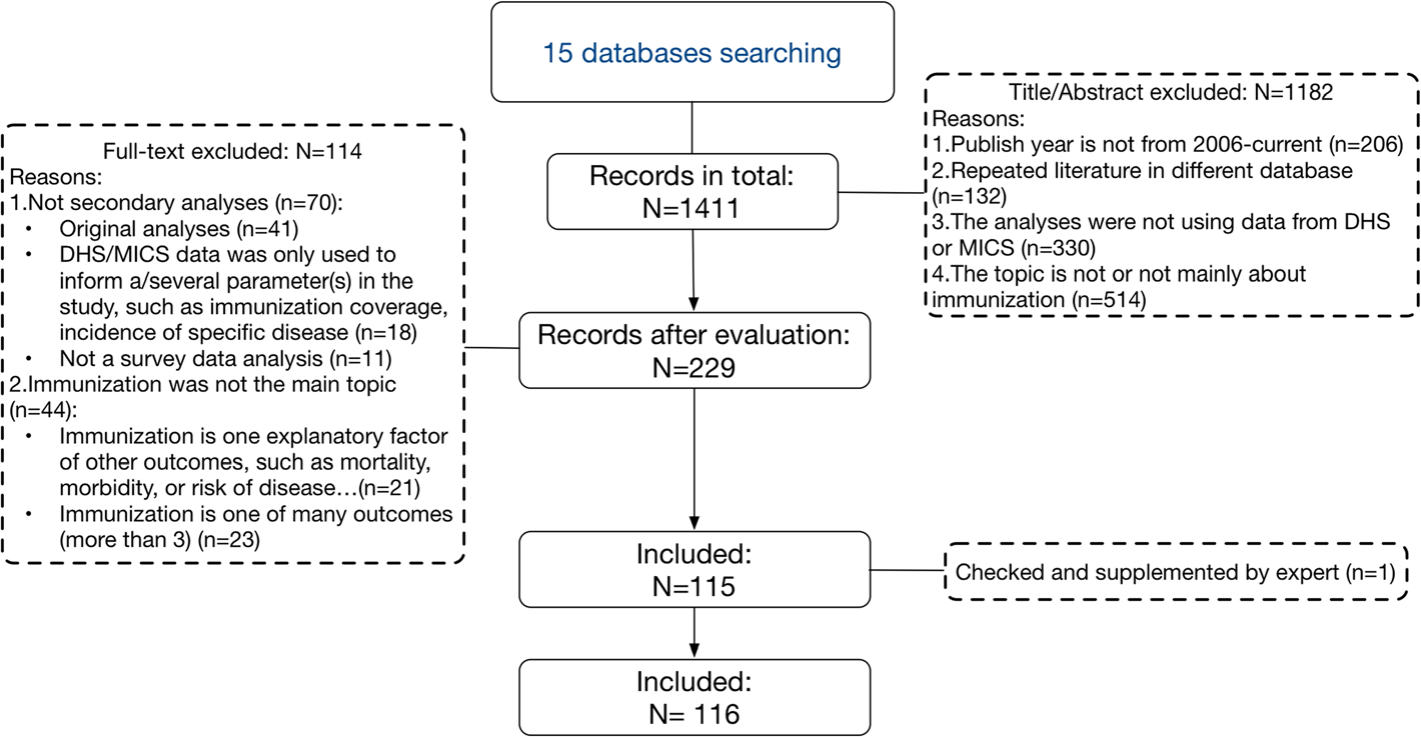
Article screening diagram

Searches in 15 database identified a total of 1411 papers, of which 229 passed title, abstract and key words content screening; 115 were eligible for our study [8, 20–133]. One additional article [134] was supplemented by the Pan American Health Organization (PAHO), serving as the WHO regional office for the Americas (Fig. 1). Thus, a total of 116 articles were included (supplement 3).

**Fig. 2:**
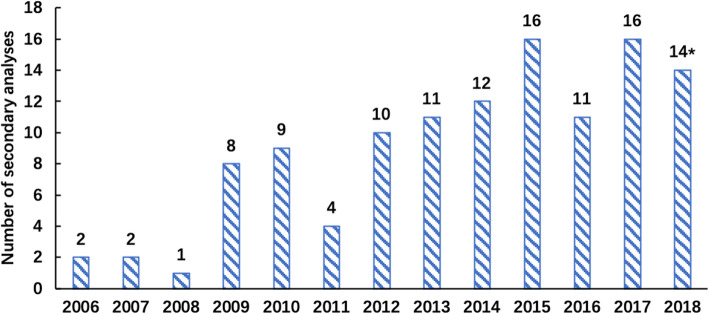
Distribution of publishing year: *Only included secondary analyses published before August 2018

Of the 116 articles included, 90 (77.6%) were published on or after 2012 and 84 (72.4%) analyses used data from a survey conducted before 2011; 66 (56.9%) analyses published within 5 years of the survey, with an average delay of 5.4 years (SD = 2.6) (Table 2). Publication of secondary immunization analyses increased overtime (Fig. 2). Regarding author affiliation, most, 80 (68.9%), of the first/corresponding authors were from high-income countries, 41 had a first or corresponding authors from the survey country, and 53 analyses had at least one of authors from the survey country. Most, 79 (68.1%), analyses used survey data from single country, and of those, only 38 (48.1%) had an author with an affiliation in that country. Nearly half of analyses conducted by high-income country used data from multiple countries.

**Table 2:** Summary of included secondary analyses

Variables	Group	Total (*N =* 116)	
		N	%
Publication metric			
Publish time period	2006–2011	26	22.4
	2012- August 2018	90	77.6
Country of affiliations^a^	High income	80	68.9
	Low-middle income	36	31.0
Country analyzed	Multiple countries	37	31.9
	Single country	79	68.1
Affiliations match with survey countries^b^	Yes	38	48.1
	No	41	51.9
Survey year (last year)	before 2011	84	72.4
	2012–2018	32	27.6
Publication delay	Mean (SD)	5.4 (2.6)	
	Within 5 years	66	56.9
	More than 5 years	50	43.1
Analyses content			
Survey types	DHS	100	86.2
	MICS	7	6.0
	Both	9	7.8
Outcomes	Specific immunization	55	41.7
	Complete/full immunization	53	40.2
	Partial or incomplete immunization	12	9.4
	Never vaccinated	11	8.3
	Other^c^	1	0.8
Type of analysis	Factors associated	77	55.4
	Inequalities	21	15.1
	Timeliness	12	8.6
	Trends	16	11.5
	Others	13	9.4
Proxies for “quality”			
How persons without documented vaccination were handled	Caregiver recall used	86	74.1
	Recall excluded	12	10.3
	Not mentioned	17	14.6
	From other sources^d^	1	0.9
Percentage of documented vaccination seen	Mentioned	36	31.0
	No mentioned	80	69.0
Weighted analysis	Yes	67	57.8
	No	4	3.4
	Not mentioned	45	38.8
Potential survey biases	Listed	54	46.6
	Not listed	62	53.4
Limitation(s) of secondary analyses	Listed	89	76.7
	Not listed	27	23.3

^a^The affiliations of first and corresponding authors separately counted; most of them (110/116) were from the same affiliation or income-level country. For those with first and corresponding author had a different affiliation (6/116), the higher income-level was used for classification

^b^Only secondary analyses using survey data from single country are included here (i.e., multi-country analyses exclude from this row)

^c^The one outcome listed as “other” was on knowledge about Human Papilloma Virus (HPV) vaccine

^d^Information from administrative activities (e.g., vaccination records kept by health facilities), censuses, and vital registration systems was referred

Most articles, 100 (86.2%), only used DHS data; 55 (41.7%) focused on a specific vaccine and 53 (40.2%) focused on complete or full immunization; 77 (55.4%) and 21 (15.1%) focused on determinants of vaccination and immunization coverage inequalities, respectively. Of the 21 studies that focused on immunization inequalities, 16 focused on socioeconomic inequalities, and 3 studies on gender-related inequalities; one study focused on geographical inequality, and one focused on both urban-rural residency and gender inequality. As per acknowledging survey or study limitations, 80 (69.0%) did not mention the percentage of card/health records seen; 17 (14.6%) did not mention how persons without documented vaccination were handled; 45 (38.8%) did not mention whether the analyses was weighted; 62 (53.4%) did not mention any potential biases; and 27(23.3%) did not mention limitations of the analyses (Table 2). There was a significant difference on specifying how persons without documented vaccination were handled between main authors from LMICs (66.7%) compared to those from high-income countries (93.8%).

To understand any trends of immunization secondary analyses, we categorized the articles by publication year (before or after 2012, the year GVAP was endorsed by the World Health Assembly). The percentage of secondary analyses mentioning potential bias and limitations increased from 23.1 to 53.3% and 50.0 to 84.4%, respectively. Other characteristic of immunization secondary analyses did not change significantly before and after 2012.

**Fig. 3:**
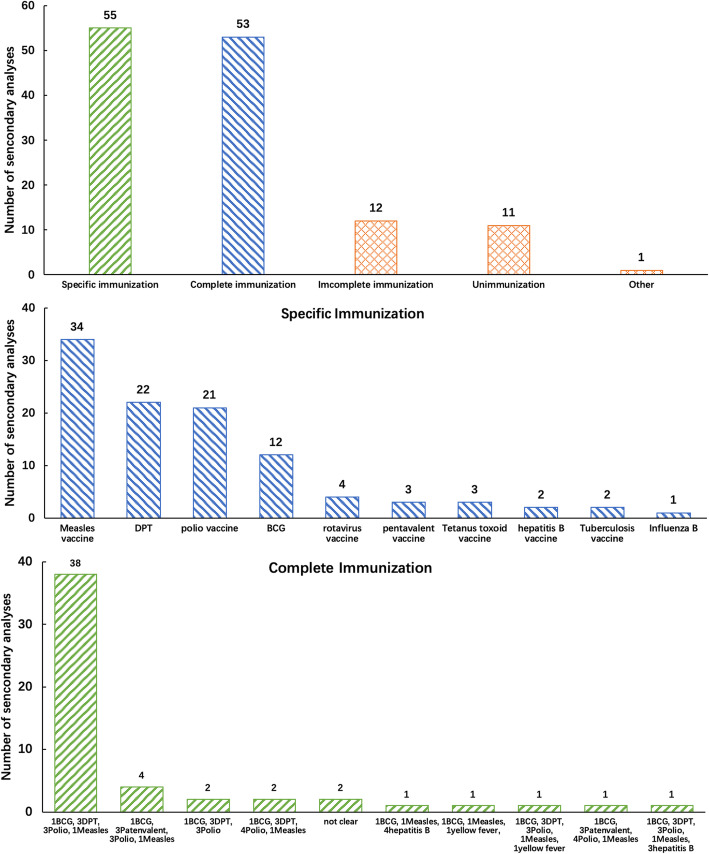
Distribution of immunization outcomes: For articles that analysed more than 1 specific immunization, all of them have been listed. Complete immunization was defined as receiving all recommended primary vaccine doses by a defined age. The third part in the figure presented the definition of complete immunization used in the different studies. Incomplete immunization was defined as not completing a set of recommended vaccine doses by a defined age. Specific immunization was defined as immunization of one type of vaccine

Figure 3 shows the distribution of immunization outcomes used of the secondary analyses included. Among all studies, 55 (41.7%) focused on specific vaccines, with measles-containing vaccines (MCV) (32.7%), diphtheria, tetanus and pertussis containing vaccines (DTP) (21.2%), and polio vaccines (20.2%) being the most frequent. 53 (40.2%) focused on complete or full immunization, mostly defined as having received one dose of BCG and three doses of DTP and one MCV dose (38 of the 53, 71.7%).

**Fig. 4:**
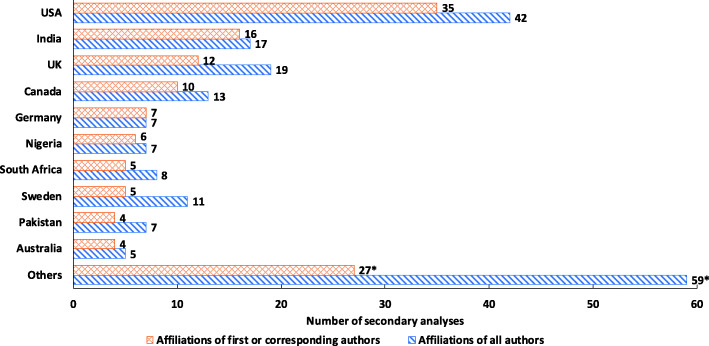
Distribution of affiliation countries: *Includes international Organizations (WHO, Gavi or UNICEF)(3/11) (affiliations of first or corresponding authors/affiliations of all authors), Brazil(3/4), Bangladesh (3/3), Ethiopia (2/3), Vietnam (2/2), Republic of Senegal (1/2), Burkina Faso (1/4), Uganda (1/3), Switzerland (1/2), Afghanistan (1/1), Belgium (1/1), China (Taiwan) (1/1), Japan (1/1), Nepal (1/1), the Netherlands (1/1), Republic of Korea (1/1), Tanzania (1/1), Côte d’Ivoire (1/1), Singapore (1/1), Kenya (1/5), Cameroon (0/2), France (0/2), Cambodia (0/1), Peru (0/1), The Democratic Republic of the Congo (0/1), Zambia (0/1), Zimbabwe (0/1), Republic of the Congo (0/1), Israel (0/1)

Figure 4 shows the distribution of affiliation countries, the top three countries contributing most of the published studies, with a first or corresponding author from that country, included the United States (US), India and the United Kingdom (UK), with 35, 15, and 12 publications, respectively. Compared with high-income countries, analyses whose main authors had a LMIC affiliation were more likely be for a single country (*P* < 0.001), to have a longer time lag between survey and publication > 5 years (*P <* 0.001), focus more on complete immunization (*P* = 0.003), and were less likely to include information on how persons without documented evidence of vaccination were handled (*P* < 0.001). No significant differences were found for other characteristic like publication year, survey type, type of analysis, mention of potential biases and the other proxies for quality (Table 3).

**Table 3:** Characterization of secondary analyses by group of affiliation countries^a^

		High-income countries (*N* = 80)		Low-middle income countries (*N* = 36)		*P*-value
		n	%	n	%	
Publication metric						
Publication year	2006–2012	20	25.0	6	16.7	0.319
	2012- August 2018	60	75.0	30	83.8	
Country analyzed	Multiple countries	34	42.5	3	8.3	< 0.001
	Single country	46	57.5	33	91.7	
Survey year	on or before 2011	54	67.5	30	83.3	0.078
	2012–2018	26	32.5	6	16.7	
Publishing delay	Mean (SD)	4.8(2.2)		6.6(3.0)		< 0.001
	Within 5 years	55	68.8	11	30.6	< 0.001
	More than 5 years	25	31.2	25	69.4	
Analysis content						
Survey types	DHS	69	86.3	31	86.1	0.979
	MICS	5	6.3	2	5.6	
	Both	6	7.4	3	8.3	
Outcomes^b^	Specific immunization	47	51.1	8	20.0	0.003
	Complete or full immunization	28	30.4	25	62.5	
	Partly or incomplete immunization	9	9.8	3	7.5	
	Never vaccinated	7	7.6	4	10.0	
	Other^b^	1	1.1	0	0.0	
Type of analysis	Factors associated	53	55.8	24	54.5	0.185
	Inequalities	12	12.6	9	20.5	
	Timeliness	9	9.5	3	6.8	
	Trends	9	9.5	7	15.9	
	Others	12	12.6	1	2.3	
Analyses quality						
Handling of persons without documented vaccination	Mentioned	75	93.8	24	66.7	< 0.001
	Not mentioned	5	6.2	12	33.3	
Percentage of card seen	Mentioned	27	33.8	9	25.0	0.346
	No mentioned	53	66.2	27	75.0	
Weighted analysis	Yes	48	60.0	19	52.8	0.807
	No	3	3.8	1	2.8	
	Not mentioned	29	36.2	16	44.4	
Potential biases listed	Yes	38	47.5	16	44.4	0.760
	No	42	52.5	20	55.6	
Limitation(s) listed	Yes	65	81.3	24	66.7	0.086
	No	15	18.7	12	33.3	

^a^Affiliation countries of first and corresponding authors were analysed here. Most of them (110/116) were from the same affiliation or income-level country, for those different (6/116) the higher income-level was used for classification

^b^The one outcome listed as “other” was knowledge about HPV vaccine

## Discussion

In our literature review, we analysed the immunization secondary analyses using DHS or MICS data since 2006 in order to better understand to what extent the DHS and MICS immunization data has been utilized beyond the coverage indicators included in the standard DHS and MICS reports. We also explored what vaccination issues were most looked at and whether some metrics related to “quality” were being included in these articles. We identified 116 published immunization secondary analyses using data from DHS and MICS, with over 3 out of 4 being published after 2012. The endorsement of GVAP by the World Health Assembly in 2012 may have been factor for the increase in secondary immunization analyses from multipurpose household surveys.

Interestingly, many more secondary analyses have used DHS data, as opposed to MICS, suggesting that the longer history of DHS database standardization and sharing may be a factor, but other factors may exist. Given that now more MICS surveys are being conducted, including sub-national surveys in many countries, it is possible that this difference will disappear going forward.

Most of the secondary immunization analyses focused on complete or full immunization, or in coverage with specific vaccines, and explored factors associated with these outcomes. Ideally, to make results more useful to immunization managers, these analyses need to be timely and be available to them. Even though DHS and MICS are only conducted in LMICs, most analyses are led by organization outside these countries; the underrepresentation of health researchers from LMICs in the scientific literature has been recently highlighted [135] and the PREPSS initiative has been born to support researchers from LMICs publish in the peer-reviewed international journals [136]. The median time delay between survey and secondary analysis publication was over 5 years. Though we did not explore the delay between survey implementation and report and dataset release by DHS and MICS, this is likely an important factor explaining this lag. Nevertheless, the time delay was worse when an organization in a LMICs contributed to the secondary analysis. Time delays and foreign authors likely reduce the usefulness of the results for immunization programmes and this should make us reflect and strategize on how to facilitate the conduction of secondary analyses immediately after DHS and MICS databases are released, and with the engagement of the country where the survey took place.

Other concerning findings are the fact that many articles did not provide information to assess the limitations of the analyses, for example how persons without documented evidence of vaccination were handled (the validity of recall has been increasingly questioned [137–139]); whether the analysis was adequately weighted; and potential bias. At least, some of these elements seem to be increasingly reported. Finally, only a small proportion of analyses focused on more immunization-specific performance analyses such as timeliness, coverage trends and missed opportunities for vaccination (MOVs), which, in general, cannot be obtained from routine immunization information systems. To support timely, quality and in-country survey immunization analyses, WHO has released a suite of programs, along with detailed documentation, to calculate vaccination coverage survey indicators that go beyond only coverage, in a documented, standardized and replicable manner; these programs are known as the Vaccination Coverage Quality Indicators (VCQI) [137, 140]. Also, several capacity-building activities related to survey analysis are ongoin g[141].

Factors related to diverse immunization outcomes and immunization inequalities were commonly assessed. Inequities are a top priority for the global immunization community and in the human health development agenda, as highlighted by the Immunization Equity Reference Group (ERG) [142], and surveys can help diagnose these issues [143].

Studies exploring inequalities looked into socioeconomic factors and analyzed individual factors such as parental education, religion, urban/rural residence, etc. Overall, the most common factors associated to lower immunization coverage include place of residence, socioeconomic status, maternal education and birth order, but other factors that have been found to affect immunization uptake, such distance to immunization centres, quality of immunization services, behaviour and attitude of health personnel cannot be assessed from DHS and MICS, as they do not collect those variables. Now, research about interventions that will actually decrease inequities and identify and reduce immunization gaps between the privileged and vulnerable populations are still lackin g[144]. Regarding timeliness of vaccination, studies considered a dose timely if the child had received it within a certain timeframe of the recommended age, but definitions varied between studies; this issue has also been noted by other author s[145].

An important limitation in household surveys like DHS and MICS is the unknown magnitude of information bias. DHS and MICS first determine immunization history by documented vaccination, usually by transcribing information from the home-based record (HBR) or vaccination card; occasionally they seek vaccination information in health facilities. If documented evidence of vaccination is missing, caregiver’s recall is used. Thus, the percentage of immunization records seen and how people without vaccination documentation are handled is important to reflect about the accuracy of the results [138, 139, 146]. In our review, though almost three quarters of the analyses (74.1%) included recall information of caregiver, 14.6% did not mention how the results from recall were handled and less than a third (31.0%) reported the specific percentage of documented vaccination seen, even as this percentage is available in all DHS and MICS reports. Thinking about other limitations of the surveys and the analyses, there is also room for improvement. Only half of analyses were explicit about the potential biases of the survey and close to 40% of the studies failed to indicate whether the analysis was weighted (as it should be to generalize the results, given the complex sampling design used by DHS and MICS). Not all is bleak; a higher percentage of articles specify bias and limitations after 2012, compared to the earlier period.

Our systematic review has several limitations. We included only DHS and MICS surveys given their standard methods and more availability of their datasets for public use. Thus, nutrition and other multipurpose surveys that also include immunization were not included. Datasets from EPI surveys are rarely made publicly available, limiting the possibility of conducting secondary analyses. Given the limited time to conduct this study, the initial article screening and data extraction were performed only by the first author (YH) with support from the senior one (MCD-H), which may have resulted in excluding relevant articles, and also non-English papers may have been missed. We did not search the EMBASE database because of inaccessibility at WHO, but we searched all 15 related available databases in WHO, especially including databases (LILACS, Popline, African Journals online, IMSEAR, WPRIM) from developing regions of Latin America, Africa, South-East Asia Region, and Western Pacific Region, which benefit the review by increasing the chance of including LMIC publications. We also contacted experts from WHO, Gavi and UNICEF for them to supplement any article we might have missed. Thus, we believe that the thoroughness of the search and review would have minimized this possibility and the main conclusions of this review stand. Further analyses of the types of journals where articles are published was not done. Also, we did not explore whether the metrics analysed have changed following the release of the revised WHO Vaccination Coverage Cluster Survey Manual in 2015 and the suite of supporting materials to conduct analyses beyond coverage thereafter. Finally, maybe our main limitation is that we did not search the grey literature [147], perhaps analyses done as thesis for university students or country immunization staff, that can potentially be more useful to immunization managers than those reported in the peer-reviewed literature.

## Conclusion

We identified over a hundred publications of secondary immunization analyses and characterised them. Most secondary analyses used DHS, rather than MICS, data and more such analyses have been done in recent years. The fact that most authors of these analyses are affiliated with institutions in high-income countries (as opposed to LMICs where these surveys are conducted), the delays between survey implementation and the publication of the analyses, in addition to some incompleteness in reporting some proxies related to the quality of the analyses illustrates that more needs to be done to build capacity to do, and publish, these analyses in LMICs and to make them useful to inform immunization programs. It is now time to better understand country awareness about the conduction of immunization secondary analyses and their use, to promote taking further advantage of the immunization component of DHS and MICS.

## Supplementary Information

**Additional file 1: Fig. S1.** Summary of DHS and MICS overtime: The grey bars on top of the blue bars refer to unavailable DHS datasets; the green bars on top of the blue bars refer to ongoing or not yet available DHS; the grey bars on top of the orange bars refer to unavailable MICS; the green bars on top of the orange bars refer to ongoing or not yet available MICS. **Table S1.** Summary of DHS and MICS by year.*. **Table S2.** Included publications (*n* = 116)*.

## Data Availability

Not applicable.

## Abbreviations

DHS: The Demographic and Health Survey
MICS: Multiple Indicator Cluster Survey
GIVS: Global Immunization Vision and Strategy
LMICs: Low and middle-income countries
PAHO: Pan American Health Organization
VPD: Vaccine-preventable diseases
GVAP: Global Vaccine Action Plan
SIA: Supplementary Immunization Activity
MOV: Missed Opportunities for Vaccination
VCQI: Vaccination Coverage Quality Indicators
HBR: Home-Based Record
