# Supplementary material for: Characterization of immunization secondary analyses using demographic and health surveys (DHS) and multiple indicator cluster surveys (MICS), 2006–2018
Source: BMC Public Health. 2021 Feb 12;21:351. doi: 10.1186/s12889-021-10364-0 (PMC7880859; doi:10.1186/s12889-021-10364-0)
Supplement: Supplementary file 1 — Additional file 1: Fig. S1. Summary of DHS and MICS overtime: The grey bars on top of the blue bars refer to unavailable DHS datasets; the green bars on top of the blue bars refer to ongoing or not yet available DHS; the grey bars on top of the orange bars refer to unavailable MICS; the green bars on top of the orange bars refer to ongoing or not yet available MICS. Table S1. Summary of DHS and MICS by year.*. Table S2. Included publications (n = 116)*. [file 12889_2021_10364_MOESM1_ESM.docx]

eFigure 1. Summary of DHS and MICS overtime: The grey bars on top of the blue bars refer to unavailable DHS datasets; the green bars on top of the blue bars refer to ongoing or not yet available DHS; the grey bars on top of the orange bars refer to unavailable MICS; the green bars on top of the orange bars refer to ongoing or not yet available MICS.


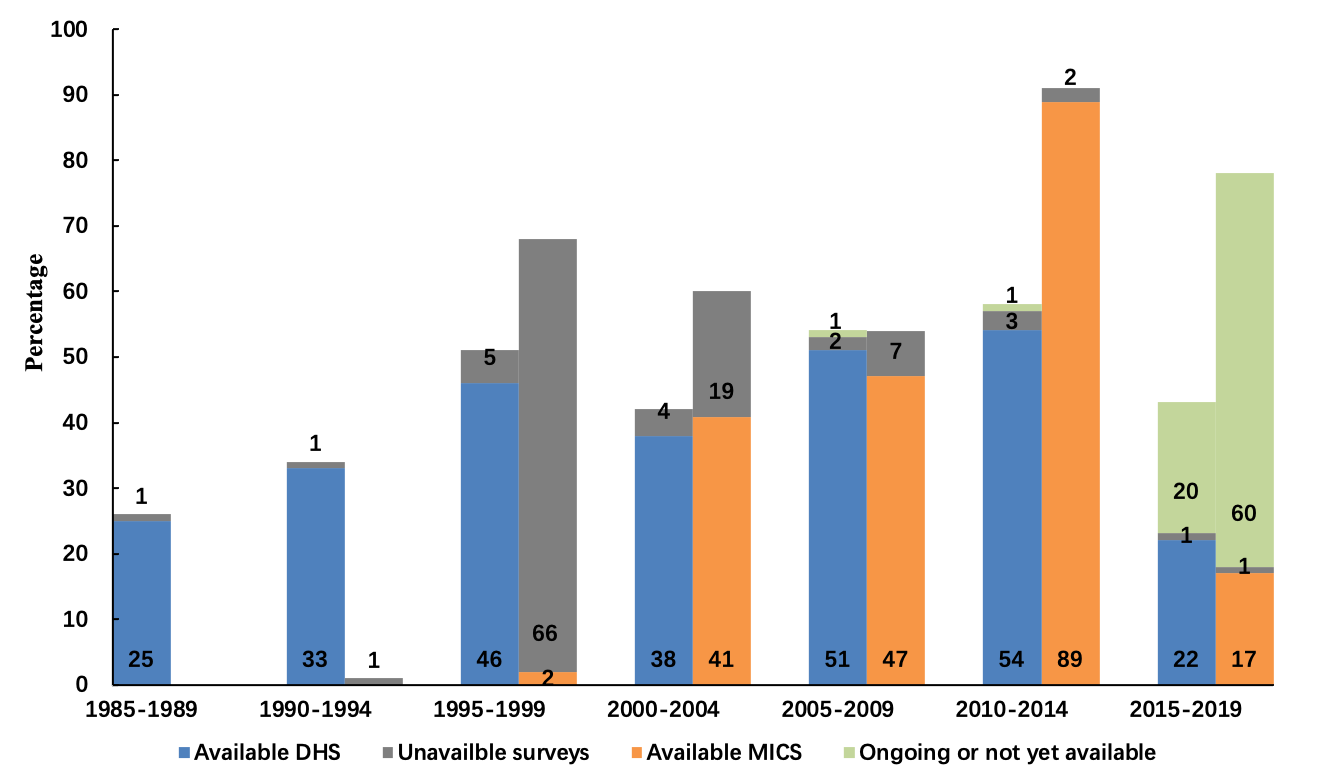


eTable 1. Summary of DHS and MICS by year.*

|  | DHS | | | MICS | | |
| --- | --- | --- | --- | --- | --- | --- |
| year | Surveys | N | N(Available)(%) | Surveys | N | N(Available) (%) |
| 1985 | El Salvador | 1 | 1(100) | - | - | - |
| 1986 | Brazil, Colombia, Dominican Republic, Liberia, Peru, Senegal | 6 | 6(100) | - | - | - |
| 1987 | Burundi, Ecuador, Guatemala, Indonesia, Mali, Mexico, Morocco, Sri Lanka, Thailand, Trinidad and Tobago | 10 | 10(100) | - | - | - |
| 1988 | Botswana, Egypt, Ghana, Togo, Tunisia, Zimbabwe | 6 | 5(83) | - | - | - |
| 1989 | Bolivia, Kenya, Uganda | 3 | 3(100) | - | - | - |
| 1990 | Colombia, Jordan, Nigeria, Paraguay, Sudan | 5 | 5(100) | - | - | - |
| 1991 | Brazil, Cameroon, Dominican Republic, Indonesia, Pakistan | 5 | 5(100) | - | - | - |
| 1992 | Egypt, Guinea, Madagascar, Malawi, Morocco, Namibia, Niger, Peru, Rwanda, Tanzania, Yemen, Zambia | 12 | 11(92) | - | - | - |
| 1993 | Burkina Faso, Ghana, India, Kenya, Philippines, Senegal, Turkey | 7 | 7(100) | Bangladesh | 1 | 0(0) |
| 1994 | Bangladesh, Bolivia, Côte d'Ivoire, Indonesia, Zimbabwe | 5 | 5(100) | - | - | - |
| 1995 | Central African Republic, Colombia, Egypt, Eritrea, Guatemala, Haiti, Kazakhstan, Uganda | 8 | 7(88) | Algeria, Bangladesh, China, Democratic Republic of the Congo, Eswatini, Ethiopia, Ghana, Islamic Republic of Iran, Kyrgyzstan, Liberia, Madagascar, Malawi, Maldives, Mozambique, Republic of the Union of Myanmar, Nigeria, Oman, Pakistan, Sierra Leone, Sudan (including current Republic of South Sudan), Syrian Arab Republic, Turkey, Turkmenistan, Zambia | 24 | 0(0) |
| 1996 | Benin, Brazil, Comoros, Dominican Republic, Mali, Nepal, Peru, Tanzania, Uzbekistan, Zambia | 10 | 10(100) | Angola, Plurinational State of Bolivia, Burkina Faso, Burundi, Central African Republic, Croatia, Cote d'Ivoire, Egypt, Gabon, Gambia, Guinea, Guinea-Bissau, India, Indonesia, Iraq, Kenya (20 of 56 districts), Lao People's Democratic Republic, Lesotho, Mali, Mauritania, Mongolia, Niger, Panama, Philippines, Sao Tome and Principe, Senegal, Somalia (Northwest Zone - Somaliland), State of Palestine (West Bank and Gaza Strip), United Republic of Tanzania, Togo, Viet Nam, Yemen, The Federal Republic of Yugoslavia (including current Serbia and Montenegro) | 33 | 0(0) |
| 1997 | Bangladesh, Chad, Egypt, Indonesia, Jordan, Kyrgyz Republic, Madagascar, Mozambique, Senegal, Vietnam, Yemen | 11 | 9(82) | Afghanistan, Islamic Republic of Iran, Nepal (Six Cycles), Somalia (Northeast Zone) | 4 | 0(0) |
| 1998 | Bolivia, Cameroon, Egypt, Ghana, Kenya, Nicaragua, Niger, Philippines, South Africa, Togo, Turkey | 11 | 10(91) | Democratic People's Republic of Korea | 1 | 0(0) |
| 1999 | Burkina Faso, Cote d'Ivoire, Dominican Republic, Guatemala, Guinea, India, Kazakhstan, Nigeria, Senegal, Tanzania, Zimbabwe | 11 | 10(91) | Georgia, The Former Yugoslav Republic of Macedonia, Nigeria, Philippines, Sudan (South), Zambia | 6 | 2(33) |
| 2000 | Armenia, Bangladesh, Cambodia, Colombia, Egypt, Ethiopia, Gabon, Haiti, Malawi, Namibia, Peru, Rwanda, Turkmenistan | 13 | 12(92) | Afghanistan (Selected Regions of East), Albania, Algeria, Azerbaijan, Bahrain, Plurinational State of Bolivia, Bosnia and Herzegovina, Botswana, Burundi, Cameroon, Central African Republic, Chad, Comoros, Cuba, Cote d'Ivoire, Dominican Republic, Equatorial Guinea, Eswatini, Gambia, Guinea-Bissau, Guyana, India, Indonesia, Islamic Republic of Iran, Iraq, Kenya, Democratic People's Republic of Korea, Lao People's Democratic Republic, Lebanon, Lesotho, Madagascar, Republic of Moldova, Mongolia, Republic of the Union of Myanmar, Niger, Rwanda, Sao Tome and Principe, Senegal, Sierra Leone, Somalia, State of Palestine (West Bank and Gaza Strip), Sudan (including current Republic of South Sudan), Suriname, Syrian Arab Republic, Syrian Arab Republic (Palestinian Refugee Camps and Gatherings), Tajikistan, Togo, Trinidad and Tobago, Tunisia, Ukraine, Uzbekistan, Bolivarian Republic of Venezuela, Viet Nam, The Federal Republic of Yugoslavia (including current Serbia and Montenegro) | 54 | 39(72) |
| 2001 | Benin, Mali, Mauritania, Nepal, Nicaragua, Uganda | 6 | 5(83) | Angola, Democratic Republic of the Congo, Lebanon (Palestinians), Maldives | 4 | 2(50) |
| 2002 | Dominican Republic, Eritrea, Jordan, Vietnam, Zambia | 5 | 4(80) | - | - | - |
| 2003 | Bolivia, Burkina Faso, Egypt, Ghana, Indonesia, Kenya, Mozambique, Nigeria, Philippines, South Africa, Turkey | 11 | 10(91) | Afghanistan, Libya | 2 | 0(0) |
| 2004 | Bangladesh, Cameroon, Chad, Lesotho, Madagascar, Malawi, Morocco | 7 | 7(100) | - | - | - |
| 2005 | Armenia, Cambodia, Cape Verde, Colombia, Congo, Egypt, Ethiopia, Guinea, Moldova, Rwanda, Senegal, Tanzania | 12 | 11(92) | Albania, Belarus, Burundi, Georgia, Jamaica, Mongolia, Tajikistan, Ukraine | 8 | 8(100) |
| 2006 | Azerbaijan, Benin, Haiti, Honduras, India, Mali, Nepal, Niger, Peru, Uganda, Zimbabwe | 11 | 11(100) | Algeria, Bangladesh, Belize, Bosnia and Herzegovina, Burkina Faso, Cameroon, Central African Republic, Cuba, Cote d'Ivoire, Djibouti, Gambia, Ghana, Guinea-Bissau, Iraq, Kazakhstan, Kyrgyzstan, Lao People's Democratic Republic, Lebanon (Palestinians), The Former Yugoslav Republic of Macedonia, Malawi, Montenegro, Sao Tome and Principe, Serbia, Sierra Leone, Somalia, Suriname, Syrian Arab Republic, Syrian Arab Republic (Palestinian Refugee Camps and Gatherings), Thailand, Togo, Trinidad and Tobago, Tunisia, Turkmenistan, Uzbekistan, Viet Nam, Yemen | 36 | 32(89) |
| 2007 | Bangladesh, Congo Democratic Republic, Dominican Republic, Indonesia, Jordan, Liberia, Namibia, Pakistan, Sri Lanka, Swaziland, Ukraine, Zambia | 12 | 12(100) | Guyana, Mauritania, Nigeria | 3 | 3(100) |
| 2008 | Bolivia, Egypt, Ghana, Nigeria, Peru, Philippines, Rwanda, Sierra Leone, Turkey | 9 | 8(89) | Ghana (District), Kenya (Eastern Province), Mozambique, Vanuatu | 4 | 2(50) |
| 2009 | Albania, Guyana, Jordan, Kenya, Lesotho, Madagascar, Maldives, Peru, Samoa, Sao Tome and Principe | 10 | 9(90) | Kenya (Mombasa Informal Settlements), Democratic People's Republic of Korea, Zimbabwe | 3 | 2(67) |
| 2010 | Armenia, Burkina Faso, Burundi, Cambodia, Colombia, Malawi, Peru, Rwanda, Tanzania, Timor-Leste | 10 | 10(100) | Bhutan, Central African Republic, Chad, Democratic Republic of the Congo, Eswatini, Gambia, Guinea-Bissau, Mali, Mongolia, Republic of the Union of Myanmar, Nepal (Mid- and Far-Western Regions), Pakistan (Balochistan), Serbia, Serbia (Roma Settlements), Sierra Leone, Republic of South Sudan, State of Palestine, Sudan, Suriname, Togo | 20 | 19(95) |
| 2011 | Bangladesh, Cameroon, Equatorial Guinea, Ethiopia, Mozambique, Nepal, Peru, Senegal, Uganda, Zimbabwe | 10 | 9(90) | Afghanistan, Belize, Costa Rica, Cuba, Ghana, Ghana (Accra), Indonesia (Papua Selected Districts), Indonesia (West Papua Selected Districts), Iraq, Jamaica, Kazakhstan, Kenya (Nyanza Province), Lebanon (Palestinians), The Former Yugoslav Republic of Macedonia, The Former Yugoslav Republic of Macedonia (Roma Settlements), Mauritania, Nigeria, Pakistan (Punjab), Somalia (Northeast Zone), Somalia (Somaliland), Trinidad and Tobago, Viet Nam | 22 | 22(100) |
| 2012 | Benin, Comoros, Congo, Cote d'Ivoire, Gabon, Guinea, Haiti, Honduras, Indonesia, Jordan, Kyrgyz Republic, Niger, Peru, Tajikistan | 14 | 14(100) | Argentina, Barbados, Belarus, Bosnia and Herzegovina, Bosnia and Herzegovina (Roma Settlements), Lao People's Democratic Republic, Madagascar (South), Republic of Moldova, Mongolia (Khuvsgul Aimag), Mongolia (Nalaikh District), Qatar, Saint Lucia, Tunisia, Ukraine | 14 | 14(100) |
| 2013 | Dominican Republic, Gambia, Liberia, Mali, Namibia, Nigeria, Pakistan, Peru, Philippines, Senegal, Sierra Leone, Turkey, Yemen | 13 | 11(85) | Algeria, Bangladesh, Montenegro, Montenegro (Roma Settlements), Panama, Thailand, Uruguay | 7 | 7(100) |
| 2014 | Bangladesh, Cambodia, Congo Democratic Republic, Egypt, Ghana, Kenya, Lesotho, Peru, Senegal, Togo, Zambia | 11 | 10(91) | Benin, Cameroon, Cuba, Dominican Republic, Egypt (Sub-national), El Salvador, Eswatini, Guinea-Bissau, Guyana, Kenya (Bungoma County), Kenya (Kakamega County), Kenya (Turkana County), Kosovo under UNSC res. 1244, Kosovo under UNSC res. 1244 (Roma, Ashkali, and Egyptian Communities), Kyrgyzstan, Malawi, Mongolia, Nepal, Oman, Pakistan (Punjab), Pakistan (Sindh), Sao Tome and Principe, Serbia, Serbia (Roma Settlements), State of Palestine, Sudan, Viet Nam, Zimbabwe | 28 | 27(96) |
| 2015 | Afghanistan, Chad, Colombia, Guatemala, Rwanda, Senegal, Zimbabwe | 7 | 7(100) | Congo, Kazakhstan, Mali, Mauritania, Mexico | 5 | 5(100) |
| 2016 | Angola, Armenia, Ethiopia, India, Malawi, Maldives, Myanmar, Nepal, Senegal, South Africa, Tanzania, Timor-Leste, Uganda | 13 | 11(85) | Belize, Côte d'Ivoire, Guinea, Mexico (Mexico City), Mongolia (Khuvsgul Aimag), Mongolia (Nalaikh District), Paraguay, Senegal (Dakar City), Thailand, Thailand (14 Provinces), Thailand (Bangkok Small Community), Turkmenistan | 12 | 10(83) |
| 2017 | Albania, Burundi, Haiti, Indonesia, Niger, Papua New Guinea, Philippines, Senegal, Tajikistan | 9 | 4(45) | Democratic People's Republic of Korea, Lao People's Democratic Republic, Nigeria, Pakistan (Gilgit-Baltistan), Pakistan (Khyber Pakhtunkhwa), Sierra Leone, Togo | 7 | 2(29) |
| 2018 | Bangladesh, Benin, Cameroon, Gabon, Guinea, Jordan, Mali, Nigeria, Pakistan, Sierra Leone, Turkey, Zambia | 12 | 0(0) | Algeria, Democratic Republic of the Congo, Costa Rica, Cuba, Gambia, Georgia, Ghana, Iraq, Kiribati, Kyrgyzstan, Lesotho, Madagascar, Mongolia, Montenegro, Montenegro (Roma Settlements), Pakistan (Balochistan), Pakistan (Punjab), Pakistan (Sindh), Suriname, Tunisia, Turks and Caicos Islands | 21 | 0(0) |
| 2019 | India, Liberia | 2 | 0(0) | Argentina, Bangladesh, Belarus, Bosnia and Herzegovina, Bosnia and Herzegovina (Roma Settlements), Central African Republic, Chad, Côte d’Ivoire, Dominican Republic, El Salvador, Equatorial Guinea, Eswatini, Fiji, Guinea-Bissau, Guyana, Honduras, Kosovo under UNSC res. 1244, Kosovo under UNSC res. 1244 (Roma settlements), Lebanon, The Former Yugoslav Republic of Macedonia, The Former Yugoslav Republic of Macedonia (Roma Settlements), Malawi, Nepal, Sao Tome and Principe, Serbia, Serbia (Roma Settlements), State of Palestine, Sudan, Thailand, Trinidad and Tobago, Turkmenistan, Uzbekistan, Zimbabwe | 33 | 0(0) |

*Country names listed as reported in the DHS and MICS websites. The names shown and the designations used on this table do not imply the expression of any opinion whatsoever on the part of the World Health Organization concerning the legal status of any country, territory, city or area or of its authorities, or concerning the delimitation of its frontiers or boundaries.

eTable 2. Included publications (n=116)*

| Publication year | First Author | Publication title | Survey type | Survey conduct year (earliest year) | Survey country |
| --- | --- | --- | --- | --- | --- |
| 2006 | Chunling Lu | Effect of the Global Alliance for Vaccines and Immunisation on diphtheria, tetanus, and pertussis vaccine coverage: an independent assessment | DHS | 2004 | 66 countries |
| 2006 | Heidi W. Reynolds | Adolescents' use of maternal and child health services in developing countries | DHS | 2001 | 15 developing countries |
| 2007 | Sudhir Anand | Health workers and vaccination coverage in developing countries: an econometric analysis | DHS | 2004 | 49 developing countries |
| 2007 | Manas K. Akmatov | Determinants of childhood vaccination coverage in Kazakhstan in a period of societal change: Implications for vaccination policies | DHS | 1999 | Kazakhstan |
| 2008 | Filip Meheus | Achieving better measles immunization in developing countries: does higher coverage imply lower inequality? | DHS | 2001 | 21 developing countries |
| 2009 | Drissa Sia | Rates of coverage and determinants of complete vaccination of children in rural areas of Burkina Faso (1998-2003) | DHS | 2003 | Burkina Faso |
| 2009 | Md. Mosiur Rahman | Tetanus toxoid vaccination coverage and differential between urban and rural areas of Bangladesh | DHS | 2004 | Bangladesh |
| 2009 | Andrew Clark | Timing of children's vaccinations in 45 low-income and middle-income countries: an analysis of survey data | DHS | 2002 | 45 low-income and middle-income countries |
| 2009 | Abel Bicaba | Monitoring the performance of the Expanded Program on Immunization: the case of Burkina Faso | DHS | 2003 | Burkina Faso |
| 2009 | Diddy Antai | Inequitable childhood immunization uptake in Nigeria: a multilevel analysis of individual and contextual determinants | DHS | 2003 | Nigeria |
| 2009 | Diddy Antai | Faith and child survival: the role of religion in childhood immunization in Nigeria | DHS | 2003 | Sweden |
| 2009 | Daniel J Corsi | Gender inequity and age-appropriate immunization coverage in India from 1992 to 2006 | NFHS(Indian DHS) | 2006 | India |
| 2009 | Jennifer N. Bondy | Identifying the determinants of childhood immunization in the Philippines | DHS | 2003 | Philippines |
| 2010 | Innocent A Semali | Trends in Immunization Completion and Disparities in the Context of Health Reforms: The case study of Tanzania | DHS | 2004 | Tanzania |
| 2010 | Mosiur Rahman | Factors affecting acceptance of complete immunization coverage of children under five years in rural Bangladesh | DHS | 2004 | Bangladesh |
| 2010 | Rathavuth Hong Æ Vathany Chhea | Trend and Inequality in Immunization Dropout Among Young Children in Cambodia | DHS | 2005 | Cambodia |
| 2010 | Slim Haddad | Heterogeneity in the validity of administrative-based estimates of immunization coverage across health districts in Burkina Faso: implications for measurement, monitoring and planning | DHS | 2003 | Burkina Faso |
| 2010 | Diddy Antai | Migration and child immunization in Nigeria: individual- and community-level contexts | DHS | 2003 | Nigeria |
| 2010 | Nicholas P. Oliphant | The contribution of Child Health Days to improving coverage of periodic interventions in six African countries | DHS and MICS | 2000 | Six African countries |
| 2010 | D. Sahu | Why immunization coverage fails to catch up in India? A community-based analysis | NFHS(Indian DHS) | 1999 | India |
| 2010 | W. Joe | Socio-economic inequalities in child health: recent evidence from India | NFHS(Indian DHS) | 2006 | India |
| 2011 | RC Fernandez | Determinants of apparent rural-urban differentials in measles vaccination uptake in Indonesia | DHS | 2007 | Indonesia |
| 2011 | Renae Fernandez | Correlates of first dose of measles vaccination delivery and uptake in Indonesia | DHS | 2007 | Indonesia |
| 2011 | Justin Lessler | Measuring the Performance of Vaccination Programs Using Cross-Sectional Surveys: A Likelihood Framework and Retrospective Analysis | DHS | 2009 | Ghana, Madagascar, and Sierra Leone |
| 2011 | S. Anil Chandran | National Family Health Survey-3 reported low full-immunization coverage rates in Andhra Pradesh, India: who is to be blamed? | DHS | 2006 | India |
| 2012 | Charles S. Wiysonge | Individual and Contextual Factors Associated with Low Childhood Immunisation Coverage in Sub-Saharan Africa: A Multilevel Analysis | DHS | 2010 | Sub-Saharan Africa |
|  |  |  |  |  |  |
| 2012 | Cesar G Victora | How changes in coverage affect equity in maternal and child health interventions in 35 Countdown to 2015 countries: an analysis of national surveys | DHS and MICS | 2008 | 35 low-income and middle-income countries |
| 2012 | Anu Rammohan | Paternal education status significantly influences infants’ measles vaccination uptake, independent of maternal education status | DHS | 2008 | Indonesia, India, Pakistan, Nigeria, Democratic Republic of Congo and Ethiopia. |
| 2012 | Manish M. Patel | Removing the Age Restrictions for Rotavirus Vaccination: A Benefit-Risk Modeling Analysis | DHS and MICS | 2009 | 158 LMICs |
| 2012 | Shanta Pandeya | Determinants of child immunization in Nepal: The role of women's empowerment | DHS | 2006 | Nepal |
| 2012 | Xavier Bosch-Capblanch | Unvaccinated children in years of increasing coverage: how many and who are they? Evidence from 96 low- and middle-income countries | DHS and MICS | 2008 | 96 low- and middle-income countries |
| 2012 | Justin Berk | The impact of a novel franchise clinic network on access to medicines and vaccinations in Kenya: a cross-sectional study | DHS | 2009 | Kenya |
| 2012 | Diddy Antai | Gender inequities, relationship power, and childhood immunization uptake in Nigeria: a population-based cross-sectional study | DHS | 2008 | Nigeria |
| 2012 | Manas K Akmatov | Timeliness of childhood vaccinations in 31 low and middle-income countries | MICS | 2007 | 31 low and middle-income countries |
| 2012 | Lucky Singh | Assessing the Utilization of Maternal and Child Health Care among Married Adolescent Women: Evidence from India | NFHS(Indian DHS) | 2006 | India |
| 2010 | Abhishek Kumar | Influence of Maternal Education on Child Immunization and Stunting in Kenya | DHS | 2003 | Kenya |
| 2013 | Carine Van Malderen1 | Decomposing Kenyan socio-economic inequalities in skilled birth attendance and measles immunization | DHS | 2008 | Kenya |
| 2013 | Wenjing Tao | Routine vaccination coverage in low- and middle-income countries: further arguments for accelerating support to child vaccination services | DHS | 2009 | 71 low- and middle-income countries |
| 2013 | Prashant Kumar Singh | Trends in Child Immunization across Geographical Regions in India: Focus on Urban-Rural and Gender Differentials | DHS | 2005 | India |
| 2013 | Kavita Singh | Maternal Autonomy and Attitudes Towards Gender Norms: Associations with Childhood Immunization in Nigeria | DHS | 2008 | Nigeria |
| 2013 | Cheryl A. Moyer | The relationship between facility delivery and infant immunization in Ethiopia | DHS | 2011 | Ethiopia |
| 2013 | Zelalem T. Haile | Determinants of Utilization of Sufficient Tetanus Toxoid Immunization During Pregnancy: Evidence from the Kenya Demographic and Health Survey, 2008-2009 | DHS | 2009 | Kenya |
| 2013 | Edward Bbaale | Factors influencing childhood immunization in Uganda | DHS | 2006 | Uganda |
| 2013 | Priyanka Dixit | Strategies to Improve Child Immunization via Antenatal Care Visits in India: A Propensity Score Matching Analysis | NFHS(Indian DHS) | 2006 | India |
| 2013 | Ravi Prakash | Urban poverty and utilization of maternal and child health care services in India | NFHS(Indian DHS) | 2006 | India |
| 2013 | Abhishek Kumar | Influence of family structure on child health: evidence from India | NFHS(Indian DHS) | 2006 | India |
| 2013 | Abhishek Singh | The Consequences of Unintended Pregnancy for Maternal and Child Health in Rural India: Evidence from Prospective Data | NFHS(Indian DHS) | 1999 | India |
| 2014 | Syed Mohammad Assad Zaidi | Coverage, timeliness, and determinants of immunization completion in Pakistan Evidence from the Demographic and Health Survey (2006-07) | DHS | 2007 | Pakistan |
| 2014 | Zachary Wagnera | Comparative performance of public and private sector delivery of BCG vaccination: evidence from Sub-Saharan Africa | DHS | 2011 | Sub-Saharan Africa |
| 2014 | B. A. Ushie | Trends and patterns of under-5 vaccination in Nigeria, 1990-2008: what manner of progress? | DHS | 2008 | Nigeria |
| 2014 | Prashant Kumar Singh | Sibling composition and child immunization in India and Pakistan, 1990-2007 | DHS | 2007 | Pakistan |
| 2014 | Stéphane Helleringer | Polio supplementary immunization activities and equity in access to vaccination: evidence from the demographic and health surveys | DHS | 2002 | 20 countries |
| 2014 | S. Clouston | Social inequalities in vaccination uptake among children aged 0-59 months living in Madagascar: an analysis of Demographic and Health Survey data from 2008 to 2009 | DHS | 2009 | Madagascar |
| 2014 | Maureen E. Canavan | Correlates of complete childhood vaccination in East African countries | DHS | 2011 | "East African Countries (Burundi, Ethiopia, Kenya, Rwanda, Tanzania, and Uganda)” |
| 2014 | Ayesha Siddiqa Bugvi | Factors associated with non-utilization of child immunization in Pakistan: evidence from the Demographic and Health Survey 2006-07 | DHS | 2007 | Pakistan |
| 2014 | Abhijeet Anand | Estimating the Likely Coverage of Inactivated Poliovirus Vaccine in Routine Immunization: Evidence From Demographic and Health Surveys | DHS | 2013 | Sub-Saharan Africa (31 countries) and South and Southeast Asia (9 countries) |
| 2014 | Oyelola A. Abegoye | Multi-year trend analysis of childhood immunization uptake and coverage in Nigeria | DHS | 2008 | Nigeria |
| 2014 | Ranjan Kumar Prusty | Socioeconomic dynamics of gender disparity in childhood immunization in India, 1992-2006 | NFHS(Indian DHS) | 2006 | India |
| 2014 | Chetna Malhotra | Maternal autonomy and child health care utilization in India: results from the National Family Health Survey | NFHS(Indian DHS) | 2006 | India |
| 2015 | Mluleki Tsawe | Factors influencing the use of maternal healthcare services and childhood immunization in Swaziland | DHS | 2007 | Swaziland |
| 2015 | A. Schweitzer1 | Improved coverage and timing of childhood vaccinations in two post-Soviet countries, Armenia and Kyrgyzstan | DHS | 2012 | Armenia and Kyrgyzstan |
| 2015 | Rodolfo Rossi | Do Maternal Living Arrangements Influence the Vaccination Status of Children Age 12-23 Months? A Data Analysis of Demographic Health Surveys 2010-11 from Zimbabwe | DHS | 2011 | Zimbabwe |
| 2015 | Elijah O. Onsomu | Maternal Education and Immunization Status Among Children in Kenya | DHS | 2009 | Kenya |
| 2015 | C. J. E. Metcalf | Transport networks and inequities in vaccination: remoteness shapes measles vaccine coverage and prospects for elimination across Africa | DHS | 2012 | 26 African countries |
| 2015 | Yihunie Lakew | Factors influencing full immunization coverage among 12-23 months of age children in Ethiopia: evidence from the national demographic and health survey in 2011 | DHS | 2011 | Ethiopia |
| 2015 | Y. Kawakatsu | Effects of three interventions and determinants of full vaccination among children aged 12-59 months in Nyanza province, Kenya | MICS | 2011 | Kenya |
| 2015 | Mohammad Hajizadeh | Paid maternity leave and childhood vaccination uptake: Longitudinal evidence from 20 low-and-middle-income countries | DHS | 2008 | 20 low-and-middle-income countries |
| 2015 | Jane O. Ebot | "Girl Power!": The Relationship between Women's Autonomy and Children's Immunization Coverage in Ethiopia | DHS | 2011 | Ethiopia |
| 2015 | Alfred Douba | Sociodemographic factors associated with incomplete immunization of children aged 12 to 59 months in six West African countries | DHS | 2012 | Six West African countries: Côte d’Ivoire, Ghana, Burkina Faso, Mali, Guinea, and Liberia |
| 2015 | Valeria Cetorelli | The impact of the Iraq War on neonatal polio immunisation coverage: a quasi-experimental study | MICS | 2011 | Iraq |
| 2015 | Silvia Bermedo-Carrasco | Predictors of having heard about human papillomavirus vaccination: Critical aspects for cervical cancer prevention among Colombian women | DHS | 2010 | Colombia |
| 2015 | Manas K. Akmatov | Evaluation of invalid vaccine doses in 31 countries of the WHO African Region | DHS | 2013 | 31 countries of the WHO African Region |
| 2015 | Samir A. Abadura | Individual and community level determinants of childhood full immunization in Ethiopia: a multilevel analysis | DHS | 2011 | Ethiopia |
| 2015 | Ranjan Kumar Prusty | Differentials in child nutrition and immunization among migrants and non-migrants in Urban India | DHS | 2006 | India |
| 2015 | Natalie McGlynn | Increased use of recommended maternal health care as a determinant of immunization and appropriate care for fever and diarrhoea in Ghana: an analysis pooling three demographic and health surveys | DHS | 2008 | Ghana |
| 2016 | A. Schweitzer | Impact of rotavirus vaccination on coverage and timing of pentavalent vaccination – Experience from 2 Latin American countries | DHS | 2012 | Honduras, Peru |
| 2016 | Richard Rheingansa | Inequalities in full immunization coverage: trends in low- and middle-income countries | DHS and MICS | 2012 | 86 LMICs |
| 2016 | Maria Clara Restrepo-Méndez | Missed opportunities in full immunization coverage: findings from low- and lower-middle-income countries | DHS and MICS | 2000 | LMICs |
| 2016 | Dadja Essoya Landoh | Predictors of incomplete immunization coverage among one to five years old children in Togo | MICS | 2010 | Togo |
| 2016 | Jennifer Lara Kriss | Vaccine receipt and vaccine card availability among children of the Apostolic faith: analysis from the 2010-2011 Zimbabwe demographic and health survey | DHS | 2011 | Zimbabwe |
| 2016 | Ahmadreza Hosseinpoor | State of inequality in diphtheria-tetanus-pertussis immunisation coverage in low-income and middle-income countries: a multicountry study of household health surveys | DHS and MICS | 2013 | 21 low-income and middle-income countries |
| 2016 | Charles C. Chima | Spillover effect of HIV-specific foreign aid on immunization services in Nigeria | DHS | 2008 | Nigeria |
| 2016 | Dao Thi Minh An | Timely immunization completion among children in Vietnam from 2000 to 2011: a multilevel analysis of individual and contextual factors | MICS | 2011 | Vietnam |
| 2016 | Saira Afzal | Effective role of lady health workers in immunization of children in Pakistan | DHS | 2013 | Pakistan |
| 2016 | Prashant Kumar Singh | Looking beyond the male-female dichotomy' - sibling composition and child immunization in India, 1992-2006 | NFHS(Indian DHS) | 2005 | India |
| 2016 | Chandan Kumar | Socioeconomic disparities in coverage of full immunisation among children of adolescent mothers in India, 1990-2006: a repeated cross-sectional analysis | NFHS(Indian DHS) | 2006 | India |
| 2017 | Olalekan A. Uthman | Children who have received no routine polio vaccines in Nigeria: Who are they and where do they live? | DHS | 2013 | Nigeria |
| 2017 | Saki Takahashi | The geography of measles vaccination in the African Great Lakes region | DHS | 2014 | 10 contiguous countries in the African Great Lakes region |
| 2017 | Emily Smith-Greenaway | Maternal migration and child health: An analysis of disruption and adaptation processes in Benin | DHS | 2006 | Benin |
| 2017 | Aparna Schweitzer | Hepatitis B vaccination timing: results from demographic health surveys in 47 countries | DHS | 2014 | 47 LMICs |
| 2017 | Peter Austin Morton Ntenda | Analysis of the effects of individual and community level factors on childhood immunization in Malawi | DHS | 2010 | Malawi |
| 2017 | Mouhamed Abdou Salam Mbengue | Determinants of complete immunization among Senegalese children aged 12-23 months: evidence from the demographic and health survey | DHS | 2011 | Senegal |
| 2017 | Mouhamed Abdou Salam Mbengue | Vaccination coverage and immunization timeliness among children aged 12-23 months in Senegal: a Kaplan-Meier and Cox regression analysis approach | DHS | 2014 | Senegal |
| 2017 | Vu Duy Kien | Trends in childhood measles vaccination highlight socioeconomic inequalities in Vietnam | MICS | 2014 | Vietnam |
| 2017 | Putri Herliana | Determinants of immunisation coverage of children aged 12-59 months in Indonesia: a cross-sectional study | DHS | 2012 | Indonesia |
| 2017 | Tenley K. Brownwright | Spatial clustering of measles vaccination coverage among children in sub-Saharan Africa | DHS | 2013 | Sub-Saharan Africa |
| 2017 | Ashish KC | Increased immunization coverage addresses the equity gap in Nepal | DHS and MICS | 2014 | Nepal |
| 2017 | Catherine Arsenault | Country-level predictors of vaccination coverage and inequalities in Gavi-supported countries | DHS | 2014 | Gavi-supported countries (45 countries.) |
| 2017 | Catherine Arsenault | Monitoring equity in vaccination coverage: A systematic analysis of demographic and health surveys from 45 Gavi-supported countries | DHS | 2014 | 45 Gavi-supported countries |
| 2017 | Sulaimon T. Adedokun | Incomplete childhood immunization in Nigeria: a multilevel analysis of individual and contextual factors | DHS | 2013 | Nigeria |
| 2017 | Muhammad Tahir Khan | Maternal education, empowerment, economic status and child polio vaccination uptake in Pakistan: a population based cross sectional study | DHS | 2013 | Pakistan |
| 2017 | Mohd Zuhair | Socioeconomic Determinants of the Utilization of Antenatal Care and Child Vaccination in India | NFHS(Indian DHS) | 2006 | India |
| 2018 | C. Edson Utazi | High resolution age-structured mapping of childhood vaccination coverage in low and middle income countries | DHS | 2013 | Cambodia, Nigeria and Mozambique |
| 2018 | Nurnabi Sheikh | Coverage, Timelines, and Determinants of Incomplete Immunization in Bangladesh | DHS | 2014 | Bangladesh |
| 2018 | Iryna Postolovska | Impact of measles supplementary immunisation activities on utilisation of maternal and child health services in low-income and middle-income countries | DHS and MICS | 2014 | 28 LMICs |
| 2018 | Allison Portnoy | Impact of measles supplementary immunization activities on reaching children missed by routine programs | DHS | 2008 | 14 LMICs |
| 2018 | Lubna A Al-Ansary | Explorations of inequality: childhood immunization | DHS | 2016 | Afghanistan, Chad, Democratic Republic of the Congo, Ethiopia, India, Indonesia, Kenya, Nigeria, Pakistan, Uganda |
| 2018 | Nina B. Masters | Vaccination timeliness and co-administration among Kenyan children | DHS | 2014 | Kenya |
| 2018 | Adrienne Kols | Provincial differences in levels, trends, and determinants of childhood immunization in Pakistan | DHS | 2012 | Pakistan |
| 2018 | Hafsa Imrana | Routine immunization in Pakistan: comparison of multiple data sources and identification of factors associated with vaccination | MICS | 2011 | Pakistan |
| 2018 | Celina M. Hanson | Enhancing immunization during second year of life by reducing missed opportunities for vaccinations in 46 countries | DHS | 2016 | 46 countries |
| 2018 | Jose Echaiz | Unintended pregnancy and its impact on childhood rotavirus immunization in Peru | DHS | 2012 | Peru |
| 2018 | Jodie Dionne-Odom | Predictors of Infant Hepatitis B Immunization in Cameroon: Data to Inform Implementation of a Hepatitis B Birth Dose | DHS | 2011 | Cameroon |
| 2018 | Matthew L. Boultona | Socioeconomic factors associated with full childhood vaccination in Bangladesh, 2014 | DHS | 2014 | Bangladesh |

*Country names listed as reported in the DHS and MICS websites. The names shown and the designations used on this table do not imply the expression of any opinion whatsoever on the part of the World Health Organization concerning the legal status of any country, territory, city or area or of its authorities, or concerning the delimitation of its frontiers or boundaries.
